# The New Territorial Force Nursing Service

**Published:** 1921-07-30

**Authors:** 


### THE MATRONS' AND SISTERS' DEPARTMENT.

### THE NEW TERRITORIAL FORCE NURSING SERVICE.

It is now generally recognised that a voluntary nursing reserve, organised on a territorial basis in conjunction with the volunteer forces of the nation, is an indispensable adjunct to the regular military nursing services. The Territorial Force Nursing Service has no longer to justify its existence. It is a body which has won its palms, and has before it the high purpose of maintaining a reputation second to none.

There are not wanting short-sighted people who depreciate the value of organisation undertaken in advance of an emergency all are concerned to avoid. These friends of hasty measures were very conspicuous in the days before war broke out, and did all in their power to stop recruiting for the service. It was their contention that the authorities would be able to secure as many nurses as they chose as soon as the need for them arose, and that no preparation beyond ordinary training was required for performing the duties of nursing sister in time of war. The event has shown the value of systematic training in military as distinct from civil nursing, and it has fully justified the decision to give a territorial basis to what is practically a war nursing reserve.

The new regulations provide for a comprehensive organisation embracing the whole of Great Britain under the Matron-in-Chief. Of the twenty-three principal matrons four are in London, four in Scotland, one in Wales, and fourteen are distributed in the provinces, according to the distribution of Territorial Force centres. Sisters and staff nurses in each locality are grouped under matrons and assistant matrons, who have the opportunity of getting a first-hand knowledge of their staffs, and are charged with the duty of forming the rolls of members which will be annually furnished to each principal matron.

The strength of the service is likely always to consist in its elasticity. It must on no account be suffered to become stationary and rigid. Sisters and staff nurses are compulsorily retired as soon as they give up nursing for as long a period as two years, or on reaching the age of fifty-five, while matrons may continue to serve up to the age of sixty. So steady is the stream which carries nurses away out of their career that it is not at all probable, even with these liberal age-limits, that the service will ever consist largely of women over forty. It needs the constant influx of the younger women to keep it what it ought to be—a reserve comprising the very best nursing strength of the country, trained under the best conditions in schools alive to the latest modern theories and practice.

Senior members are liable to be called on periodically to put in a period of training in a military hospital at Army pay and allowances according to rank, and it is possible that some may be deterred by this regulation from offering themselves. That such a provision is essential to the unity and value of the force is at once apparent, and the more zealous will eagerly embrace the opportunity of enlarging their experience by gaining insight into a system of administration conspicuously distinct from civilian methods.

The important point is for the requirements of the whole service to be brought before nurses in hospital during and after their training. Hospital nurses are not admitted without the consent of their matrons, but the country looks for much more than consent from the influential heads of institutions. There should be an immediate and spontaneous response to the new regulations, which are, in effect, an appeal for recruits.

